# Development and Evaluation of a Thermosensitive In Situ Gel Formulation for Intravaginal Delivery of *Lactobacillus gasseri*

**DOI:** 10.3390/pharmaceutics14091934

**Published:** 2022-09-13

**Authors:** Ye Zhao, Tianyuan Wang, Ziyi Chen, Hao Ren, Ping Song, Yingying Zhu, Shan Liang, Chimeng Tzeng

**Affiliations:** 1 School of Pharmaceutical Sciences, Nanjing Tech University, Nanjing 211816, China; 2 School of Food and Pharmaceutical Engineering, Nanjing Normal University, Nanjing 210000, China; 3 Translational Medicine Research Center-Key Laboratory for Cancer T-Cell Theragnostic and Clinical Translation, School of Pharmaceutical Sciences, Xiamen University, Xiamen 361005, China; 4 Xiamen Chang Gung Hospital Medical Research Center, Xiamen 361022, China

**Keywords:** *Lactobacillus gasseri*, thermosensitive, vaginal gel, poloxamer, fructooligosaccharide, hyaluronic acid

## Abstract

In situ administration of vaginal probiotics has been proposed as an effective prevention strategy against gynecological diseases caused by microecological disorders. In this study, a thermosensitive in situ gel formulation was prepared for intravaginal delivery of *Lactobacillus gasseri* *(L. gasseri)*. The optimized formulation was characterized for the rheological properties, in vitro release properties, and microencapsulation efficiency. The mixtures of poloxamer 407 (26.0% *w*/*w*) and 188 (9.0% *w*/*w*) produced an increase in gelation extent at 37 °C after dilution in simulated vaginal fluid (SVF). The presence of a low concentration of hyaluronic acid (HA, 0.3% *w*/*w*) improved the mucoadhesive properties and the capability to gel at 37 °C. Additionally, the viability of *L. gasseri* encapsulated with alginate or via co-extrusion technique with fructooligosaccharide (FOS, 0.5% *w*/*w*) was maintained at 11 log CFU/mL for eight weeks at 4 °C. In conclusion, the evaluation of the in situ thermosensitive gel formulation was shown to be efficacious for intravaginal delivery of *L. gasseri* with suitable textural and rheological properties.

## 1. Introduction

Within the vagina, the intense interaction between a variety of microorganisms and hosts provides the first line of defense against the migration of opportunistic pathogens. Under healthy conditions, the microbiome maintains a stable balance known as eubiosis. When the microecological balance is disrupted, known as dysbiosis, the pathogenic microorganisms continue to multiply and cause vaginal-related diseases [1]. Women with abnormal proliferation of anaerobic bacteria are at an increased risk of sexually transmitted infections, such as human immunodeficiency virus (HIV), *Mycoplasma genitalium*, human papillomavirus (HPV), and herpes simplex virus (HSV), etc. [2,3,4,5,6]. Although antibiotics have been proven to be an effective treatment strategy in eliminating bacterial pathogens in the short term, relapse of infection, further dysbiosis, and side effects remain serious problems [7].

In situ administration of lactobacillus has a great potential to restore vaginal health, as the vaginal microflora of healthy women is typically dominated by one of the four Lactobacilli species: *Lactobacillus* *gasseri* (*L. gasseri*), *Lactobacillus crispatus* (*L.* *crispatus*), *Lactobacillus iners* (*L. iners*)*,* and *Lactobacillus jensenii* (*L. jensenii*) [8]. *L. gasseri* has been approved by European Food Safety Authority (EFSA) and listed as a microorganism suitable for use in food and feed production [9]. As a member of the Gram-positive lactobacillus genus, *L. gasseri* protects the vaginal epithelial barrier from pathogen colonization and invasion by producing antimicrobial peptides, lactic acid, and hydrogen peroxide [10] and adhering to vaginal epithelial cells (VECs) to form a protective film to competitively inhibit pathogen adhesion [11]. *L. gasseri* E has been demonstrated to effectively prevent vaginal bacterial infections through direct inhibition of pathogen colonization in the vaginal lumen [12]. Moreover, pretreatment of *L. gasseri* JM1 attenuated the lipopolysaccharide-induced inflammatory response as evidenced by the downregulation of genes encoding proinflammatory cytokines and the upregulation of genes encoding anti-inflammatory cytokines [13]. The clinical isolation of *L. gasseri* LG1 was identified to have the potential to inhibit vulvovaginal candidiasis (VVC) by anti-adhesion and anti-biofilm formation [14]. *L. gasseri* HMV18 from a healthy vagina could inhibit the growth of food-borne pathogens such as *Escherichia coli* (*E. coli*), *Salmonella enterica* (*S. aureus*), and *Klebsiella oxytoca* (*K. oxytoca*) [15]. Moreover, the vaginal administration of *L. gasseri* LN40 resulted in vaginal colonization, resulting in lower recurrences and less malodorous discharge [16]. In general, the use of *L. gasseri* for the vaginal administration could be a practical strategy for vaginal disease prevention. Therefore, *L. gasseri* was selected in the present study as the therapeutically effective probiotic for topical replenishment of vaginal microflora. 

Local and direct vaginal administration has been investigated as a more effective route of drug delivery due to the accessible surface area of the vaginal mucosa with rich blood supply, avoidance of gastrointestinal tract disturbance, and hepatic first-pass metabolism [17]. However, effective vaginal administration is challenged by the high variability in anatomy, physiology, and microbiology in addition to the presence of secretions and vaginal fluids that reduce the residence time of the formulation [18]. In this regard, in situ hydrogels have been proposed as a more convenient topical dosage form that tolerates the changes of the microenvironment and has good mucoadhesion, retention, and flowing properties to spread over the vaginal mucosa [19]. The application of polymers in hydrogel formulations has attracted extensive attention, and among these promising polymers, poloxamer compositions are suitable for vaginal gel formulations own to their thermosensitive self-assembly characteristics, sufficient resistance to dilution in presence of vaginal fluids, low biotoxicity, low stimulation for organisms, and good biocompatibility [20]. The low viscosity of the formulation consisting of poloxamers is beneficial for the drug delivery to the vaginal cavity and spreading on the mucosa at room temperature of 25 °C. An increase of the temperature from 25 °C to 37 °C promotes the in situ gelation of the administered formulation, thereby resisting the removal process of the vaginal environment, which in turn ensures long-term retention of the loaded therapeutic agent at the infection site. Poloxamers have been approved by U.S. Food and Drug Administration (FDA) and listed as pharmaceutical excipients in the United States and European Pharmacopoeias [21]. Poloxamer-based thermosensitive vaginal gel has been used in clinics as the delivery agent of *L. gasseri* (ATCC 33323) [22] and *L. crispatus* (ATCC 33197) [20]. Vaginal gel formulation with poloxamer 407 (P407), poloxamer 188 (P188), and polycarbophil-based delivery of clotrimazole were found to significantly prolong the antifungal activity of clotrimazole in vivo [23]. 

Additionally, the lack of adhesion is the main drawback of poloxamer, which can be improved by other adhesive additives [24]. Hyaluronic acid (HA) is a glycosaminoglycan that can be used as a humectant and adhesive to improve the physicochemical properties of the gel by incorporation into poloxamer gels [25]. The addition of HA to the poloxamer temperature-sensitive gel is able to slow down gel erosion, alter the microstructure of the gel, and make the gel more compact with smaller pore sizes, thereby improving the performance of the gel [25]. 

Given the aforementioned premises, this study aims to develop an in situ thermosensitive gel formulation for intravaginal delivery of *L. gasseri*. Firstly, the mixtures of P407 and P188 were assessed as the basic gel matrix for *L. gasseri* in order to achieve the suitable thermosensitive property. In addition, the gel formulation was optimized, and the release of the formulation in vitro was simulated using rheology and viscoelasticity at 25 °C before administration and at 37 °C with the dilution in simulated vaginal fluid (SVF) to mimic the in vivo administration. In the next step, the viability and stability of probiotic lactobacillus was improved by the adjunction of prebiotics and encapsulation. Moreover, the long-term stability during storage was further evaluated.

## 2. Materials and Methods

### 2.1. Chemicals

P407 and P188 were purchased from Kolliphor, BASF SE, Ludwigshafen, Germany. HA, lactose, glucomannan, lactulose alginate, and soluble starch were obtained from Shanghai Maclean Biochemical Technology Co., Ltd., Shanghai, China. Lactic acid, urea, acetic acid, and glucose were acquired from Sinopharm Chemical Reagent Co., Ltd., Shanghai, China. Bovine serum albumin was purchased from Shanghai Huixing Biochemical Reagent Co., Ltd., Shanghai, China. Fructooligosaccharide (FOS) was provided by Shanghai Yuanye Bio-Technology Co., Ltd., Shanghai, China. Syto9 and propidium iodide (PI) were offered by KeyGEN Bio TECH, Nanjing, China. 

### 2.2. Microorganism and Cultivation

*Lactobacillus gasseri* M01 GDMCC60781 (*L. gasseri* M01) was a laboratory-conserved strain that was deposited in GDMCC (Guangdong Microbial Culture Collection Center, Guangzhou, China). *L. rhamnosus* ATCC7469, *L. delbrueckii* ATCC11842, *L.* *reuteri* ATCC23272, and *L. jannaschii* ATCC25258 were purchased from American Type Culture Collection (ATCC, Rockville, MD, USA). *Lactobacillus* *plantarum* (*L. plantarum*) NRRL B-14768 was acquired from ARS Culture Collection (NRRL, Peoria, IL, USA). Lactobacillus strains were grown in Man Rogosa Sharpe (MRS) broth at 37 °C.

### 2.3. Freeze-Drying Procedure

The overnight cultures of lactobacillus were centrifuged (5000 rpm, 10 min, 4 °C) and washed twice with sterile water. The lactobacillus was resuspended in 5% (*w*/*v*) skimmed milk. The emulsion with lactobacillus was frozen overnight in at −80 °C freezer and then freeze-dried using freeze drier (SCIENTZ-10ND, Xinzhi Freeze Drying Equipment Co., Ningbo, China) for 48 h (h). The freeze-dried lactobacillus powders were stored in a sealed container and immediately analyzed for the post-processing viability, and then, the storage trials commenced. 

### 2.4. Preparation of the Thermosensitive Gel Formulations Containing L. gasseri 

The vaginal thermosensitive gel formulations of *L. gasseri* with poloxamers were prepared through cold method. Lactic acid/sodium lactate buffer (0.01 M, pH 3.2) was cooled to 4 °C. Poloxamers, HA, prebiotics, and freeze-dried bacterial powder (prepared in Section 2.3) were then slowly added to the lactic acid/sodium lactate buffer with continuous agitation. *L. gasseri* was added at the concentration of 11 log CFU/mL. The gels were left at 4 °C until a clear solution was obtained. A final pH of 3.8 was determined. Blank gels were prepared according to the above process without adding *L. gasseri*.

### 2.5. Preparation of Hydrogels Diluted by Simulated Vaginal Fluid

SVF was prepared according to the reported with 3.5 g/L NaCl, 1.4 g/L KOH, 0.2 g/L Ca(OH)_2_, 0.02 g/L bovine serum albumin, 2.0 g/L lactic acid, 1.0 g/L acetic acid, 0.2 g/L glycerol, 0.4 g/L urea, and 5.0 g/L glucose [26]. This mixture was adjusted to a pH of 4.2 using HCl and NaOH. Then, 9 mL of each of the abovementioned formulations were diluted with 1 mL of SVF.

### 2.6. Characterization of Gels

An aliquot (1 mL) of each gel was put into a glass tube. The tube was kept in cold water and then gradually heated to 37 °C at a speed of 0.5 °C/min. The flowability of the gels in the tube was observed with frequent flip-flopping. The gelation temperature was defined as the temperature at which the state of complete gelation was achieved. The final gel was placed in a water bath at 37 °C, and the gelation time was defined as the time at which the state of complete gelation was reached. 

### 2.7. Response Surface Methodology (RSM)

RSM model was applied to simultaneously optimize the levels of variables to attain the best system performance to make statistical predictions. Design Expert 8.0.6 software (Stat-Ease, Inc., Minneapolis, MN, USA).was applied to formulate a suitable poloxamer-based hydrogel. To optimize the preparation variables with the gelation temperature of resulting hydrogel, the central composite design (CCD) was applied for systemic study of joint influence of the effect of independent variables. During the preparation process, P407, P188, HA, and pH were chosen as four variables. Their levels and ranges were listed in Table 1.

To optimize the best condition to release the *L. gasseri* from the poloxamer-based hydrogel, Design Expert 8.0.6 software was applied. The CCD consists of 2 independent variables with 5 replications and center points, yielding 13 experiments in total (Table 2). The ranges of 24% to 30% P407 and 6% to 11% P188 were used according to the four-factor experiment. Formulations with suitable gelation temperatures were obtained from the above optimization. The volume of lactic acid/sodium lactate buffer (0.01 M, pH 3.2) of 10 mL was kept constant during the tests. Design Expert 8.0.6 software showed the optimum ratio of the mixture required to obtain the optimum gelation temperature of the hydrogels.

### 2.8. Rheological Characterization of the Hydrogels

The rheological properties of the gel were tested by the Anton-Paar’ s Modular Compact Rheometer 302 (MCR) rotational rheometer equipped with a cone-plate geometry. The samples were heated at a rate of 1 °C every 60 s (s) and the temperature changed from 25 °C to 45 °C during the procedure with angular frequency omega of 100 rad/s. The complex viscosity, the shear storage/elastic modulus (G’), and the shear loss/viscous modulus (G’’) were recorded as a function of the temperature. The gelation temperature was determined as the sample exhibited a switch from G” > G’ to G’ > G” [27].

### 2.9. Assessment of the Distribution of L. gasseri within the Gel

The distribution and viability of *L. gasseri* in the gel were assessed by using fluorescent stains. The fluorescent dye Syto9 stains both living and dead bacteria green, whereas PI stains dead bacteria red only [28]. Shortly, working solutions were prepared by adding 20 µL of Syto9 stock solution (1 mM) and 30 µL of PI (20 mM) to 1 mL of sterile-filtered water. The working solutions were added to the gel at a ratio of 1/1000 (*v*/*w*) and mixed thoroughly by pipetting up and down. The mixed samples were incubated in the dark at 37 °C for 30 min. Fluorescence was microphotographed under a 485 nm wavelength excitation using a fluorescence microscope (Nikon ECLIPSE Ti2E, Tokyo, Japan).

### 2.10. In Vitro Release of L. gasseri

A non-film dissolution method was used to evaluate *L. gasseri* release from gels [29]. Gel (5 mL) was added into the SVFs (10 mL) in a 25 mL glass tube and oscillated at 50 rpm and 37 °C in the dark. At predetermined time points, an aliquot (3 mL) of supernatants was withdrawn, and preheated fresh SVFs were supplemented. The gels were maintained at the bottom of the tube. The number of bacteria in the supernatants was recorded.

The mechanism of *L. gasseri* release was investigated using log-transformation and least squares regression analysis, and the data generated from the study were fitted to a power-law equation (known as the Korsmeyer–Peppas equation):

(1) $$M_{t}/M_{\infty }=k\;t^{n}$$

*M_t_/M*_∞_ is the fraction of drug released at the time *t*, *k* is the constant related to the structural and geometrical features of the delivery system, and *n* is the release exponent.

### 2.11. Evaluation of Different Prebiotics Added into the Gel

MRS broth was reconstituted to 0.5% prebiotic-supplemented dextrose-free media. Then, 1 mL of an overnight culture of each LAB strain was inoculated in 100 mL of the above-mentioned media and cultured at 37 °C. OD600 and pH were measured to indicate whether prebiotics were fermented and if this process could restore acidity.

### 2.12. Microencapsulation of L. gasseri

Calcium alginate beads of *L. gasseri* were prepared by emulsification/external gelation technique. The probiotic was prepared for the microencapsulation, and the propagated culture was harvested after around 12 h by centrifugation process (10,000 rpm, 5 min), washed twice, and resuspended in sterile physiological saline until the encapsulation step. The applied initial cell counts were between 9 and 11 log CFU/mL. The alginate (2%, 3%, 4%, 5% (*w*/*v*)) and alginate (2% (*w*/*v*)) + soluble starch (2% (*w*/*v*)) were chosen as the encapsulation material. All solutions used for entrapment were sterilized using an autoclave (at 121 °C for 15 min). Probiotic bacteria in 1 mL physiological saline were added to the encapsulation liquid (9 mL) and dispersed evenly by gentle agitation. Afterwards, 10 mL alginate–cell mixture was subsequently emulsified in 50 mL liquid paraffin containing 0.5% (*v*/*v*) Span 85 under magnetic agitation at 200 rpm for 30 min. The gelation process was started by adding 10 mL of 0.05 M calcium chloride (CaCl_2_) solution slowly and evenly along the wall of the cup into the emulsion. During one hour of continuous stirring, alginate microcapsules were gradually formed. The oil phase and the water phase were separated using a separatory funnel. The target beads were located in the water phase. The beads were collected after gentle centrifugation at 1000 rpm for 5 min.

### 2.13. Morphology and Particle Size Measurements

Particle size was measured in de-ionized water. Immediately after microencapsulation, the morphology of the probiotic-filled calcium alginate microbeads was examined with a light microscope ensuring uniform microbead shape and size distribution.

### 2.14. Stability Studies of the Gel

The gels with the addition of prebiotics or treated with alginate (3%, 4%, 5% (*w*/*v*))-encapsulated bacteria were stored over eight weeks at 4 °C and 25 °C, respectively, in order to assess their stability. *L. gasseri* distribution and viability were evaluated using fluorescent microscope. As mentioned before, the bacterial activity was illustrated by staining with Syto9 and PI.

### 2.15. Statistical Analysis

Statistical analysis was made with GraphPad Prism Version 8.0.1 (GraphPad Software, San Diego, CA, USA). The difference of the two groups was verified by Student’s *t*-test, and the difference of multiple groups was defined through one-way analysis of variance (ANOVA) followed by Tukey’s multiple comparison test. All data were presented as mean values ± standard deviation (SD). Differences were considered statistically significant at *p* < 0.05.

## 3. Results

### 3.1. Effect of Hydrogels Composition on Gelation Temperature

**Figure 1:**
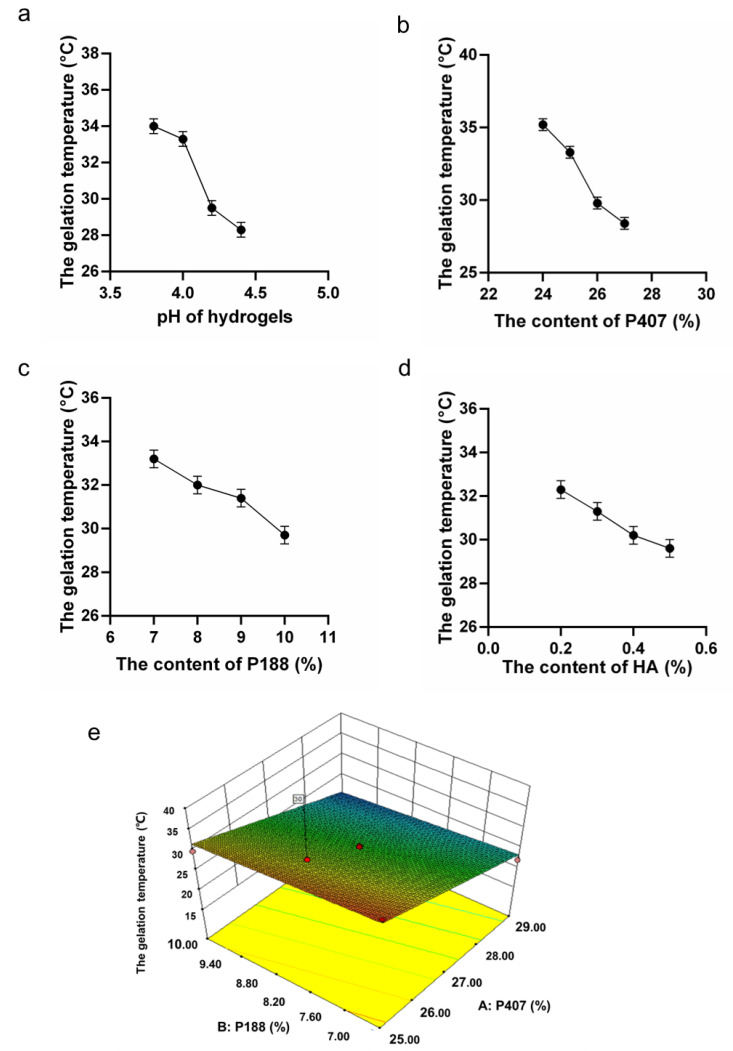
Formulation optimization by RSM test. (**a**). The lactic acid/sodium lactate buffer’s (0.01 M) pH effect on the gelation temperature (Mean ± SD, *n* = 3). (**b**). The effect of P407 on the gelation temperature. (**c**). The effect of P188 on the gelation temperature. (**d**). The effect of HA on the gelation temperature. (**e**). The 3D image of the response surface optimization model.

According to the preliminary experiments on polymer formulation, the range of poloxamer mixing with P407 was stated as 24% to 30%. The percentage of P188 ranging from 6% to 11% was proved to form the hydrogel sufficiently. Considering the maintenance of suitable adhesion and moisturizing effect of the gel, 0.3% HA was also chosen [27,30,31] (Mayol et al. 2008; Ur-Rehman et al. 2011; Nappi et al. 2021). In the bacterial infection sites, there was a growth in vaginal pH (>4.5) [32]. Keeping gel formulations at a stable pH of 3.8 was beneficial for maintaining of normal vaginal pH value. In this study, the gelation temperature of *L. gasseri* hydrogels showed to decrease with the increasing of pH value (Figure 1a). Moreover, with the increase of P407, P188, and HA, the gelation temperature also decreased, respectively (Figure 1b–d).

### 3.2. RSM Model and Target Formulation

RSM model was applied to make statistical predictions through fitting with experimental data by polynomial equation. According to the Design Expert software, an optimization choice could be applied to solve problems of optimal formulations to obtain the desired hydrogel gelation temperature of 30 °C. The experimental design and statistical analysis are shown in Table 2. The mathematical model is shown in Equation (2) (below) and Figure 1e.

(2) The gelation temperature of the gel (℃)=+27.58−5.52×P407 (%)−1.42×P188(%)

The Equation (2) and Figure 1e suggested by the software indicated the change of the gelation temperature of the *L. gasseri* hydrogel in relation to the content of poloxamers. In Table 3 and Figure 1e, the regression analysis with an R^2^ = 0.9405 indicated a satisfactory correlation between the independent variables and the response. An ANOVA test was applied to evaluate the adequacy of the empirical second-order polynomial model (Table 3); the F-value of 79.10 (*p* < 0.0001) implied that the model was significant. In the present study, P407 and P188 were identified as significant model terms. Three-dimensional surfaces and contours (Figure 1e), as the graphical representations of the regression equation, showed the considerable influences of P407 and P188 on the gelation temperature. The gelation temperature decreased smoothly with the increase of P188 from 6.0% to 11.0% and the increase of P407 from 24.0% to 30.0%.

The optimal formula was suggested by the software according to the optimal gelation temperature (30 °C) (Table 4). The experiment was carried out to validate the gelation temperature of the formulation. The formula with the composition of P407 (26%) and P188 (8.86%) as well as the tested gelation temperature are shown in Table 4. The difference between the gelation temperature obtained in the solvent and the predicted gelation temperature was less than 15%, indicating that the optimization process of the computational design was feasible. After the diluted in SVF, the gelation temperature of the formulation was lower than 37℃, implying that the formulation could be used for further analysis.

### 3.3. Rheological Characterization

**Figure 2:**
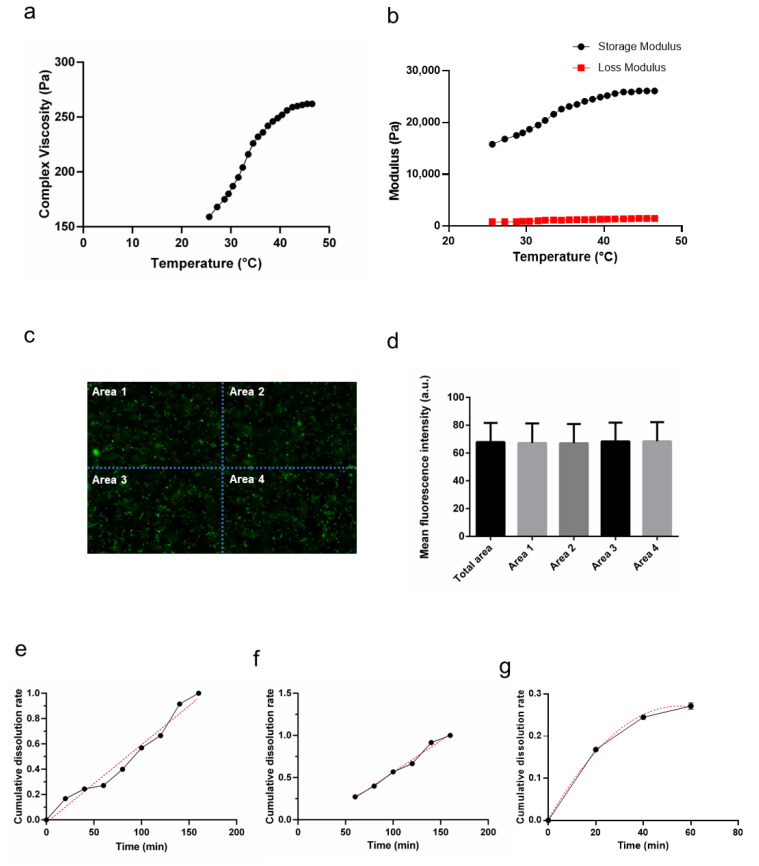
Physical–chemical properties of the gels. (**a**). The complex viscosity of the gels. (**b**). Flow curves at elevated temperatures from 25 °C and 46 °C. (**c**). The representative image of the distribution of *L. gasseri* within the gel and a schematic representation of the zoning. (**d**). The mean fluorescence intensity analysis of the regions in Figure 2C (Mean ± SD, *n* = 3). (**e**). The release curve and the simulated fitted line with a nonlinear fit to the entire curve. (**f**). The plot of nonlinear fit to the points of the release curve after 60 min and the fitted line. (**g**). The plot of a nonlinear fit to the points of the release curve before 60 min and the fitted line.

Further investigation of the rheological properties of the gel revealed the changes in the complex viscosity, storage, and modulus of loss of the gel with the alteration of temperatures, as shown in Figure 2a and b. With the increase of temperature, the viscosity of the gel gradually improved (Figure 2a), and the difference between the storage modulus and the loss modulus constantly increased (Figure 2b), indicating that the gel had a high sensitivity to the change of temperature. At 37 °C, the complex viscosity reached a relatively high level (Figure 2a), which was beneficial because the gel could be retained at the site of administration for a longer period to prolong the residence time of *L. gasseri* in the vaginal cavity. The storage modulus was significantly higher than the loss modulus of the gel (Figure 2b), suggesting that the gel exhibited elastic behavior predominantly.

### 3.4. Distribution of L. gasseri within the Gel

The Syto9 and PI stains were considered to be the effective indicators in the evaluation of cell metabolic activity and membrane integrity [33]. The distribution of *L. gasseri* was essentially the same along the x and y axes as observed in the gel (Figure 2c). The images were divided into four parts, and the mean fluorescence intensity of each part was counted separately (Figure 2c). ANOVA test confirmed the results and showed that there was no significant difference between the four parts (*p* > 0.05, Figure 2d). The results confirmed that the distribution of *L. gasseri* in the gel is uniform, which might be beneficial for the uniform distribution of *L. gasseri* in vaginal administration.

### 3.5. In Vitro Release of L. gasseri

The release curve of *L. gasseri* in Figure 2e shows that *L. gasseri* cells of the gel released continuously for 160 min until the gel was completely dissolved. The obtained data were fitted to the equations described in Section 2.10, which simulated the presumed release process and principles. The overall curve was analyzed using a nonlinear fit method with an R-squared of 0.9798 (Figure 2e). Separate nonlinear fitting of the curves would obtain a better fit. Particularly, it was showed that the linear fitting after 60 min had a better curve fit with an overall R-squared of 0.9862 (Figure 2f). The fitting formula was as follows:

(3) $$\begin{array}{c} \frac{M_{t}}{M_{\infty }}=k\;t^{n}=0.001191\;t^{1.332} \end{array}$$

*M_t_*/*M*_∞_ is the fraction of drug released at time *t*; *k* is a constant, related to the structural and geometrical features of the delivery system; *n* is the release exponent.

The nonlinear fitting before 60 min had a better curve fitting with an overall R-squared of 0.9944 (Figure 2g). The fitting formula was as follows:

(4) $$\begin{array}{c} \frac{M_{t}}{M_{\infty }}=k\;t^{n}=0.04854\;t^{0.4259} \end{array}$$

The release process could be divided into two stages with 60 min as the node. The slope of the curve gradually decreased in the first 60 min, indicating a sudden release phenomenon, and the subsequent release rate slowed down; 60 min later, the slope of the curve was more stable, and the release rate was more constant. The release curve indicated that the sustained-release properties of the gel were remarkable, which facilitated the colonization of *L. gasseri* in vivo.

### 3.6. Fermentation Profiles of Prebiotics by Vaginal Microbes

**Figure 3:**
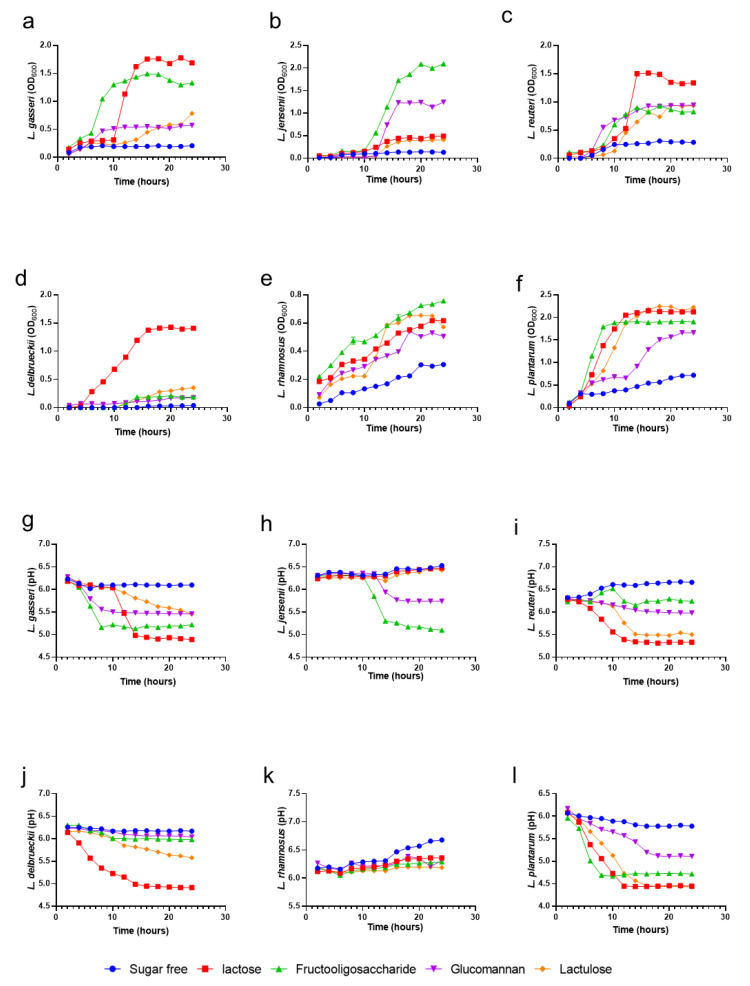
Growth curves and pH changes of vaginal lactobacilli cultured with different prebiotics. (**a**–**f**). Growth curves of various probiotics in medium with different prebiotics. (**g**–**l**). The pH changes of various probiotics in medium with different prebiotics.

Prebiotics were selected for their beneficial effects on *L. gasseri* activity in the hydrogel. Here, we compared the effects of different prebiotics on various vaginal beneficial microbes and on the maintenance of a proper pH. In the growth curve experiments with different prebiotics in Figure 3a–f, it is shown that lactose increased the maximal growth of *L. gasseri* (*p* < 0.0001), *L. jannaschii* (*p* < 0.0001), *L. reuteri* (*p* < 0.0001), *L. delbrueckii* (*p* < 0.0001), *L. rhamnosus* (*p* < 0.0001), and *L. plantarum* (*p* < 0.0001) compared to the media without prebiotic. FOS, glucomannan, and lactulose also increased the maximal growth of all the experimental strains (*p* < 0.0001). For pH maintenance tests (Figure 3g–l), lactose and galactose made the pH-lowering effect of *L. jannaschii* less significant, with *p*-values of 0.0017 and 0.0001. During the fermentation process, except for *L. rhamnosus*, the augmented acidity of the medium was directly related to the increment in the growth rate of the lactobacilli tested, which can effectively maintain normal acidic vaginal pH. Considering the effects of lactose intolerance [34] as well as the effect of lowering pH and the combination of multiple probiotic growth conditions, the use of FOS as the prebiotics was a practical solution.

### 3.7. Morphology and Particle Size of Microspheres

**Figure 4:**
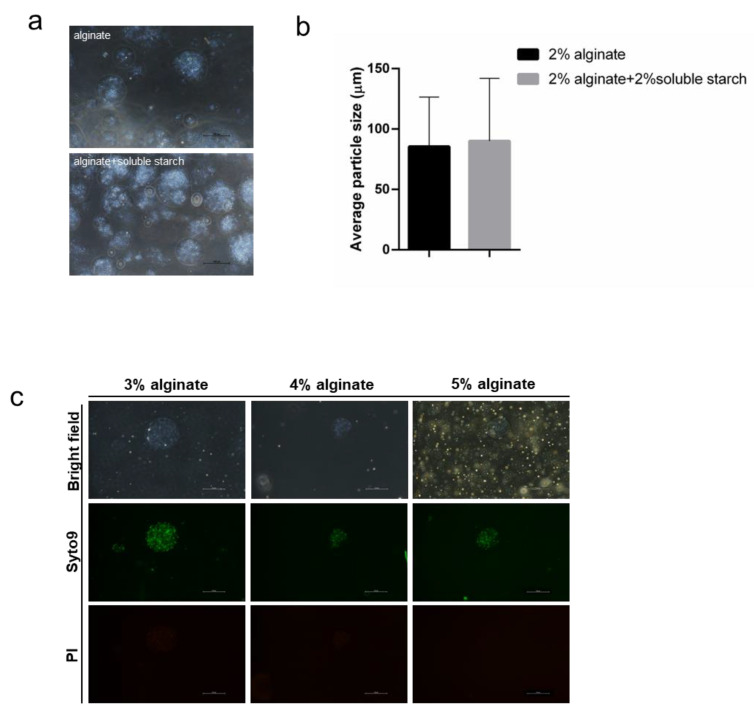
Morphology and mean particle size of the microspheres. (**a**). Morphology of sodium alginate microspheres embedded with *L. gasseri*. (**b**). Morphology of sodium-alginate-soluble starch composite microspheres encapsulated with *L. gasseri*. The scale bar corresponds to 100 µm. (**c**). Effect of encapsulant on the size of droplet with *L. gasseri*. The scale bar corresponds to 100 µm.

Microencapsulation has been shown to be an effective method to enhance bacterial activity. Microcapsules are generally spherical under the microscope (Figure 4a). The size of the distributed microbeads had a wide range, between approximately 20–220 µm. The addition of soluble starch had no significant effect on the morphological particle size of the *L. gasseri*-embedded spheres (*p* > 0.5, Figure 4b). The beads’ size enlarged as the concentration of sodium alginate in 0.05 M calcium chloride solution increased. The augmented alginate concentration led to higher viscosity, resulting in larger microspheres and greater entrapment of bacteria, thus causing a high degree of cross-linking [35]. Moreover, *L. gasseri* showed high activity at the experimental sodium alginate concentrations of 3%, 4%, and 5% (Figure 4c).

### 3.8. Influence of the Storage Conditions

**Figure 5:**
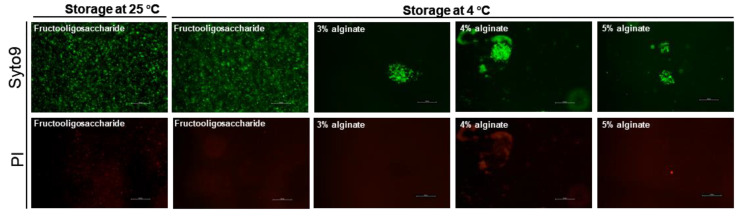
Viability of *L. gasseri* contained in the gel. The concentration of the encapsulant sodium alginate was 3%, 4%, and 5%. Living and dead bacteria were stained with Syto9 (green), and dead bacteria were stained with PI (red). The scale bar corresponds to 100 µm.

To investigate the storage conditions, the gels with FOS were stored at 4 °C and 25 °C, respectively, for eight weeks. There was no significant decrease in bacterial activity, while extremely few bacterial mortalities occurred when the gels were stored at 25 °C (Figure 5). The results suggested that the addition of probiotics could provide protection of activity similar to encapsulation. However, for the gels with alginate-encapsulated *L. gasseri*, 2% sodium alginate fragmented after eight weeks, while other concentrations (3% and 4%) of encapsulated spheres were likely to precipitate after a long-term storage. Therefore, it was preferable to add probiotics for strain protection.

## 4. Discussion

In recent years, researchers have realized that vagina is an underutilized route for drug administration. The local administration of vaginal probiotics, especially lactobacilli, are proposed as an effective prevention strategy against vaginal infectious diseases. N’Guessan Gnaman et al. developed a thermo-sensitive hydrogel by evaluation of the rheological properties and stability, which contains 21.5% of poloxamer 407, 1% of sodium alginate, and 9 log CFU of *L. crispatus* per gel sample (5 g) [20]. Vigani et al. developed a mucoadhesive in situ gelling formulation for the vaginal administration of *L. gasseri* with the main mixture of two thermosensitive polymers, i.e., P407 and methylcellulose (MC), containing 6 log CFU/mL of *L. gasseri.* The rheological properties and the cytocompatibility towards the HeLa cell line were assessed in their work [22]. In Oerlemans’ study, *L. plantarum WCFS1, L. pentosus KCA 1*, and *L. rhamnosus GG* were formulated in an innovative silicone gel at a dose of 9–10 log CFU/g. The lactobacilli remained viable over multiple months of storage at 5 °C and 25 °C. Moreover, the formulation was proven to be effective on VVC in clinic [36]. The thermosensitive gel formulation was evidenced to enhance the viability of epithelial cells without affecting the morphology of the vaginal mucosa [23]. In this study, a thermosensitive in situ gel formulation was developed for intravaginal delivery of *L. gasseri*. The final gel consisted of P407 at 26%, P188 at 8.86%, HA at 0.3%, FOS at 0.5%, and *L. gasseri* at 11 log CFU/mL. *L. gasseri* was proven to stay highly active for at least eight weeks in the formulation at 4 °C. This vaginal gel has potential as an adjuvant medication or daily prevention for vaginal infection by maintaining and restoring vaginal microecology.

Optimizing the pharmaceutical characterization of vaginal gels is essential in optimizing safety, efficacy, and acceptability. In the current work, the model for formulation optimization was established to obtain the most optimal composition and proportions; then, the optimized formulation was characterized for the rheological properties and in vitro release properties. Overall release behavior is important for a therapeutic formulation, which can be predict by mathematical models fitted by measuring some physical parameters and experimental release data. However, there is still a lack of study about the in vitro release of bacteria in the in situ gel solutions. In this research, the release was shifted from diffusion as the main driving force to swelling and relaxation of the polymer-blend matrix. The Korsmeyer–Peppas equation was applied in the piecewise fitting of release curves, and the “n” value in Equation (1) predicts the mechanism of drug release. Likewise, 0.45 ≤ n corresponds to the Fickian diffusion mechanism, 0.45 < n < 0.89 corresponds to non-Fickian transport, *n* = 0.89 corresponds to Case II transport, and n > 0.89 corresponds to super case II transport. In the first half of the fitting, “n” value was 0.4259 (<0.45), indicating that the release of bacteria in the gel acted as the Fickian transport, and the high initial osmolarity difference led to a high release rate of *L. gasseri*, which was slowly reduced due to the decrease in osmolarity. This was consistent with the vancomycin-release mechanism [37]; the fitting “n” value of the second half was 1.332 (>0.89), indicating that the release of bacteria in the gel was attributed to the super case II transport. This was consistent with the drug release trend of metformin HCl from seed mucilage (FSM)-alginate mucoadhesive beads [38]. In all, the sustained-release properties of the gel indicated by the release curve facilitated the delivery of *L. gasseri* into vaginal cavity.

To further improve the viability of the lactobacillus, the addition of prebiotics and microencapsulation were applied. It was shown that the viabilities of *L. gasseri* encapsulated with alginate and via co-extrusion technique with FOS were stably maintained for eight weeks at 4 °C, which suggested that the addition of probiotics could provide protection of activity similar to encapsulation. However, in the 2% alginate-encapsulated gel, sodium alginate fragmented after eight weeks. In 3% and 4% alginate-encapsulated gels, as the particle size increased, the microspheres precipitated after standing and did not form a good suspension in the gel. The addition of prebiotics to the formulation was significantly better than encapsulation in the improvement of the stability of the gel. Therefore, the addition of prebiotics was preferred for protection of the viability and stability of lactobacillus. In addition, the gel prepared with FOS exhibited improved storage stability compared to that reported [20]. Nevertheless, the homogeneity of the gel, the appropriate embedding method to improve the storage stability, and the stability of the bacteria in the gel still need to be further explored.

FOS is one of the most widely studied and reported prebiotics. FOS had become a probiotic functional food supplement with GRAS (Generally Recognized as Safe) status. FOS fermentation produces short-chain fatty acids and other organic acids that lower the pH of the lumen and maintain a healthy vaginal environment [39]. FOS addition can increase the viscosity and viscoelasticity of the gel [40]. In addition, FOS is able to improve the activity of lactobacillus during storage [40]. Therefore, in this study, *L. gasseri* was encapsulated by coextrusion technique with FOS (0.5% *w*/*w*) to enhance the viability.

In this study, there was a certain difference between the gelation temperature detected by the test-tube-inversion method and the rheometer test. The rheometer test results showed that the gelation temperature of the formulation was less than 25 °C, while the test-tube-inversion result was above 30 °C. This apparent discrepancy may be due to the fact that the inversion method was based on the observation of the flow state of the gel, while the rheological method determined the sol-gel transition temperature by calculation. The end points of the two methods for determination of gel state were inconsistent. Even so, it was reported that the gelation temperature obtained by the tube inversion method was positively correlated with the rheometer temperature [41]. Figure 2b showed that the storage modulus at 25° was significantly higher than the loss modulus, indicating that there was a great tendency to gelation at 25 °C after long-term storge. In the formula design, the gelation was expected to occur after administration. Therefore, based on the rheological results, 4 °C was chosen as the preferred storage temperature.

The influence of HA on the gelation properties of poloxamers blends has been well-studied. HA addition affected the self-assembly process of the poloxamer and was able to significantly enhance the rheological properties of poloxamers, thus suggesting that HA might interact with the micelles through secondary bonds (e.g., hydrogen bonds) to enhance the gel structure [27]. In addition, HA and sodium lactate buffers have a significant effect on the gelation temperature by modulating the hydrogen bonding between the polymers. It was reported that the addition of HA delayed the gelation temperature by a few degrees Celsius [27]. The same trend was also confirmed in the gel of this study. The mucoadhesion experiments demonstrated that the rheological synergy between poloxamer/HA gels lead to the changes in flow behavior from a rather Newtonian solution alone to a pseudoplastic mixture. Moreover, the strong interaction between HA-P407 and mucin enhanced the adhesion of the tissue [30]. Furthermore, HA also provided moisturizing effects [31], which was beneficial for vaginal administration.

In conclusion, the evaluation of in situ thermosensitive gel formulations was shown to be efficacious for intravaginal delivery of *L. gasseri*, with suitable textural and rheological properties. Further mucoadhesion experiments and pharmacological experiments are needed to verify the safety and effectiveness of the gel.

## Figures and Tables

**Table 1:** The actual value of design variables.

Formulation	Low Actual	High Actual
P407 (%)	24	27
P188 (%)	7	10
HA (%)	0.2	0.5
pH	3.8	4.4

**Table 2:** Complete design layout corresponding runs.

Formulation	P407 (%)	P188 (%)	Mean (±SD) Gelation Temperature (°C)
F1	25.00	7.00	35.3 ± 0.5
F2	27.00	8.50	28.4 ± 0.5
F3	27.00	6.38	29.5 ± 0.5
F4	24.17	8.50	34.2 ± 0.5
F5	27.00	10.62	27.5 ± 0.5
F6	27.00	8.50	28.1 ± 0.5
F7	27.00	8.50	28.2 ± 0.5
F8	29.00	10.00	19 ± 0.5
F9	29.83	8.50	20 ± 0.5
F10	27.00	8.50	28.3 ± 0.5
F11	25.00	10.00	29.8 ± 0.5
F12	27.00	8.50	28.2 ± 0.5
F13	29.00	7.00	22 ± 0.5

**Table 3:** The model parameters.

Source	Sum of Squares	F Value	*p*-Value Prob > F
Model	260.05	79.10	***
P407	244.00	148.45	***
P188	16.04	9.76	*
Residual	16.44		
Lack of Fit	16.39	210.07	***
Pure Error	0.052		
Cor Total	276.48		

*p*-Value < 0.001, ***; 0.01 < *p*-value < 0.05, *.

**Table 4:** The predicted and actual value of optimized desired formulation.

P407 (%)	P188 (%)	Desirability Gelation Temperature (°C)	Mean (±SD) Actuality Gelation Temperature (°C)
26	8.86	30	29.6 ± 0.5

## Data Availability

All data generated or analyzed during this study are included in this published article.
